# Digital Cognitive‐Motor Occupational Training with QEEG Monitoring in Schizophrenia Rehabilitation: An Exploratory Neurofunctional Study

**DOI:** 10.1155/oti/1389594

**Published:** 2026-03-13

**Authors:** Supalak Khemthong, Maliwan Rueankam, Winai Chatthong

**Affiliations:** ^1^ Division of Occupational Therapy, Faculty of Physical Therapy, Mahidol University, Nakhon Pathom, Thailand, mahidol.ac.th

**Keywords:** cognitive-motor training, digital health intervention, motor relearning, occupational therapy, quantitative electroencephalography, schizophrenia

## Abstract

**Introduction:**

Current occupational therapy assessments for schizophrenia rely primarily on behavioral observation, with limited access to objective neurofunctional indicators of cognitive–motor engagement. Digital tools combined with neurophysiological monitoring offer new opportunities to address this gap. This study examined a digital cognitive‐motor occupational training application paired with quantitative electroencephalography (QEEG) to explore neurofunctional engagement during task performance.

**Methods:**

Thirty individuals with schizophrenia completed structured occupational tasks under two conditions: with and without QEEG‐based Brain Mapping Performance (BMP). Task engagement was characterized using participant‐prioritized daily activities grouped into dopaminergic, serotonergic, oxytocinergic, and endorphin‐related behavioral domains, conceptualized as theoretical behavioral associations rather than direct neurochemical measures. QEEG indices included theta relative power, beta relative power, and theta/beta ratio recorded from six frontal and temporal scalp sites.

**Results:**

Theta relative power was higher at frontal and central sites (Fz and Cz) during BMP‐monitored conditions, reflecting executive and motor planning demands. Beta relative power varied across memory‐ and emotion‐related task phases, indicating task‐dependent modulation of cognitive effort. Engagement Intensity Scores were consistently higher in the unmonitored condition, while BMP monitoring was associated with longer task completion times and increased neurofunctional engagement.

**Conclusion:**

QEEG‐guided occupational task performance reveals distinct, task‐dependent neurofunctional patterns in schizophrenia rehabilitation. The integration of digital task‐based assessment with neurophysiological monitoring provides exploratory neurofunctional indicators that may support individualized, functionally informed occupational therapy interventions.

## 1. Introduction

Rehabilitation in schizophrenia increasingly recognizes the need to integrate cognitive and motor training within meaningful activities. Deficits in executive functioning, emotional regulation, and behavioral initiation impair individuals’ ability to engage in purposeful occupations [1, 2]. Occupational therapy interventions must therefore address not only observable performance skills but also the underlying neurocognitive processes that support occupational engagement [3, 4].

The Brainy2Blessly (B2B) application was developed as a dual‐purpose tool for assessing and training cognitive‐motor functions [5]. It engages users in structured digital tasks simulating daily occupations while collecting performance‐based indicators such as task completion time, memory recall, and emotional self‐report [6]. When paired with Brain Mapping Performance (BMP)—a task‐based application of quantitative electroencephalography (QEEG)—the system enables real‐time monitoring of theta activity in frontal and central brain regions, which have been associated with attentional control and motor planning [7, 8].

In the era of digital health and sensor‐based assessment, the standardization of algorithmic tools and wearable technologies has become increasingly important to ensure data quality, reproducibility, and clinical interpretability. Recent methodological frameworks emphasize transparent reporting, context‐specific validation, and cautious interpretation when integrating digital platforms into rehabilitation and performance‐monitoring settings. In particular, hypothesis‐driven designs remain essential when digital tools are applied in clinical disciplines such as occupational therapy, where functional relevance and interpretability are prioritized over purely data‐driven prediction models [9].

Within this context, wearable technologies and artificial intelligence–assisted monitoring have been increasingly applied to analyze movement patterns, cognitive–motor performance, and neurophysiological signals. Recent reviews highlight the growing role of sensor‐based systems and AI‐supported analytics in biomechanics, rehabilitation, and performance monitoring, while also underscoring the importance of aligning technological sophistication with the clinical goals of the target population. These developments provide a relevant framework for situating digital task‐based tools combined with neurophysiological monitoring, such as the present integration of a cognitive–motor occupational training application with task‐based quantitative electroencephalography [10].

This hybrid approach integrates neuroscience‐based monitoring with performance‐based assessment, supporting investigation of the relationship between cortical activity and occupational behavior in schizophrenia rehabilitation. Emerging models integrating neurophysiological indicators with observable functional performance advocate for the use of brain‐behavior data to inform individualized intervention planning and deepen understanding of cognitive‐emotional dysfunctions in everyday occupational functioning [11–13].

In the present study, BMP was employed as a neurofunctional assessment tool to examine changes in theta power, beta power, and theta/beta ratio across six frontal and temporal QEEG sites while participants engaged in memory‐ and emotion‐related occupational tasks. The study aimed to determine whether performance using the B2B application [5] under monitored versus unmonitored conditions would reveal differences in task completion time, QEEG dynamics, and perceived calmness, reflecting variations in executive function engagement.

Contemporary occupational therapy in schizophrenia increasingly integrates digital technologies with occupation‐based principles to support cognitive‐motor engagement and functional participation [14]. Thai executive function (EF) rehabilitation frameworks, such as the Neuro‐Functional Approach (NFA), conceptualize recovery as a graded process of relearning everyday routines through cognitive prompting, emotional regulation, and meaningful participation [14–16]. Within this theoretical context, the B2B application was designed as a task‐structuring tool that operationalized these principles through stepwise, occupation‐focused simulations targeting key EF domains.

The activity‐domain framework used within the B2B tasks [5] was informed by Breuning’s descriptive model of motivation and habit formation [17]; however, this model is employed strictly as a conceptual heuristic to support participant engagement and task organization, rather than as a neurophysiological or explanatory framework. Accordingly, the present study does not assume direct neurochemical specificity but treats activity domains as functional and experiential categories consistent with occupational therapy perspectives on motivation, routine formation, and affective readiness.

Moreover, the integration of B2B with real‐time QEEG via BMP supports therapy approaches that emphasize humanized cueing, environmental familiarity, and emotional readiness rather than isolated cognitive performance [2, 17]. This combined framework may enhance ecological validity and promote client‐centered care, which are critical for achieving functional gains in schizophrenia rehabilitation.

## 2. Methods

### 2.1. Participants

Thirty individuals diagnosed with schizophrenia (mean age = 38.9 ± 7.15 years; 10 male, 20 female; all right‐handed) were recruited. Inclusion criteria included DSM‐5 diagnosis and stable outpatient status. All participants were assessed using the Thai version of the Positive and Negative Syndrome Scale (PANSS) prior to EEG recording to ensure clinical stability. Individuals who exhibited acute psychotic symptoms or high positive symptom scores were excluded from the session. The PANSS Thai version has demonstrated acceptable criterion validity and interrater reliability in local populations [18]. Exclusion criteria involved comorbid neurological disorders or recent substance use. All participants provided written informed consent by signing a consent form before participating in the study.

### 2.2. Human Research Ethics

Ethical clearance was granted by Mahidol University Central IRB (MU‐CIRB 2020/114.1505; COA No. 2020/103.0708) and the Department of Mental Health IRB (DMH.IRB 031/2563; COA No. 041/2563).

### 2.3. Study Process

B2B Task Conditions EEG signals were recorded using a QEEG system during Brain Mapping Performance (BMP) tasks, allowing functional interpretation of brain activity in context‐specific B2B tasks [5]. Each participant completed two B2B task sessions: one with BMP and one without. To examine how physical activity (PA) engagement relates to emotional‐cognitive processing in individuals with schizophrenia, the B2B application integrated a classification of 16 daily activities, each theoretically associated with one of the four motivation‐affect activity domains, derived from Breuning’s descriptive model and used as a structural heuristic rather than a neurochemical classification [17]. Although activities were grouped for structural clarity, these occupational tasks likely recruit overlapping motivational, affective, and neurocognitive processes rather than isolated neurotransmitter systems.

Participants were asked to prioritize these 16 activities based on their frequency of participation in daily life. For each activity domain, a subset of four activities was presented, grounded in occupational therapy literature and neuropsychological associations:

• Dopamine‐related (reward, goal achievement): e.g., cooking, playing games, solving puzzles, planning a trip
• Oxytocin‐related (social bonding, trust): e.g., group singing, giving compliments, storytelling, sharing meals
• Serotonin‐related (emotion regulation, mindfulness): e.g., mindful walking, gardening, meditation, journaling
• Endorphin‐related (pleasure, pain relief): e.g., jogging, dancing, laughing, stretching

Although everyday occupations such as cooking or sharing meals may plausibly engage multiple motivational, social, and regulatory processes, each activity in the present study was assigned to a single domain for analytic consistency. Domain assignment was based on the primary functional intent emphasized during task selection (e.g., goal completion, social interaction, emotional regulation), rather than assuming exclusive neurochemical activation. This approach allowed standardized comparison across participants while acknowledging the multidimensional nature of occupational engagement.

The top four participant‐selected activities were matched with their respective energy expenditure values (METs) based on the Thai Physical Activity Guideline (2020) [19]. A normalized score was then computed for each activity domain by summing the MET values of the top four activities within each domain and dividing by the maximum reference value (5.9 METs). A higher score reflects the selection of activities associated with greater relative energy expenditure and should therefore be interpreted as an index of engagement intensity rather than successful habit acquisition, routine consistency, or task quality. The Engagement Intensity Score reflects normalized energy expenditure and should be interpreted as an index of engagement intensity rather than behavioral mastery or habit formation. These individualized scores were used as behavioral proxies for relative engagement intensity across activity domains conceptually associated with reward, social affiliation, emotional regulation, and pleasure, rather than as indicators of neurochemical activity.

### 2.4. Outcome Measures and Statistical Analysis

#### 2.4.1. QEEG Recording and Analysis

QEEG signals were recorded using a 12‐electrode setup based on the international 10–20 system. Data were sampled and analyzed offline to extract theta relative power (4–7 Hz), calculated as the proportion of total spectral power, from six primary electrode sites: Fp1, Fz, F4, F7, Cz, and T3. Standard bandpass filtering and artifact rejection procedures were applied. The theta/beta ratio was calculated as an index of attentional regulation, with higher ratios indicating reduced attentional control and lower ratios reflecting more focused attention. Individual alpha peak frequency (IAF) was not controlled for in the present analysis.

#### 2.4.2. Statistical Analysis

Statistical analyses were conducted using a hypothesis‐driven, site‐specific analytical approach consistent with occupational therapy assessment practices. Repeated‐measures analysis of variance (ANOVA) was employed to examine within‐subject differences in predefined QEEG indices across task conditions and electrode sites, prioritizing clinical interpretability and functional relevance. While machine learning and deep learning (ML/DL) approaches offer powerful tools for analyzing high‐dimensional and non‐linear EEG data, their application typically requires large datasets and data‐driven modeling frameworks. Given the exploratory nature of the present study and the modest sample size, advanced ML/DL techniques were not applied. Instead, traditional inferential statistics were selected to support transparent, interpretable comparisons aligned with clinical decision‐making. Future studies with larger datasets will incorporate ML/DL methods to enhance pattern detection and predictive modeling [9, 10].

## 3. Results

### 3.1. Theta and Beta Dynamics Across QEEG Sites During Cognitive Engagement

Descriptive statistics for theta relative power, theta/beta ratio, and beta relative power across six electrode sites during the memory task are presented in Table 1. Theta relative power was highest at midline frontal and central regions (Fz and Cz), suggesting increased executive and motor planning engagement. In contrast, beta relative power was comparatively elevated at T3, indicating region‐specific modulation of cognitive and auditory‐linguistic processing. The theta/beta ratio was highest at Fz, consistent with enhanced attentional regulation during task performance. Due to differences in measurement scale between relative power and ratio metrics, no inferential statistical comparisons were conducted across QEEG index types.

**Table 1:** Descriptive Statistics of QEEG Indices Across Six Electrode Sites.

QEEG Sites	Theta Relative Power (Mean ± SD)	Theta/Beta Ratio (Mean ± SD)	Beta Relative Power (Mean ± SD)
Fp1	18.21 ± 5.70	1.32 ± 0.58	13.80 ± 7.44
F7	17.83 ± 4.05	1.18 ± 0.47	15.11 ± 6.93
F4	20.12 ± 4.99	1.14 ± 0.51	17.65 ± 9.03
Fz	22.77 ± 6.04	1.39 ± 0.67	16.38 ± 9.01
Cz	20.85 ± 5.51	1.14 ± 0.55	18.29 ± 10.06
T3	14.40 ± 4.72	0.68 ± 0.36	21.18 ± 13.19

Theta relative power during B2B was descriptively highest at Fz, followed by Cz, and F4, consistent with engagement of frontal and central regions commonly associated with executive and cognitive‐motor processing. These regional patterns suggest increased attentional and motor planning demands during task performance. Beta relative power appeared comparatively elevated at T3, whereas Fz maintained the highest theta dominance, indicating region‐specific variations in cortical activation patterns. The theta/beta ratio was also highest at Fz, consistent with enhanced attentional regulation during task engagement.

### 3.2. Impact of BMP Guidance on Activity‐Domain Engagement Intensity

As shown in Table 2, engagement intensity scores across four activity domains were compared under conditions with and without BMP guidance. Participants showed consistently higher engagement intensity scores in the unmonitored (No BMP) condition across all activity domains, including reward‐related (t_29_ = ‐3.179, p = 0.013), emotion‐regulation (t_29_ = ‐1.317, p = 0.011), social affiliation (t_29_ = ‐2.418, p = 0.022), and pleasure‐oriented domains (t_29_ = ‐3.291, p = 0.012). For descriptive purposes, the strongest correlations (p <0.05) between QEEG indices and activity‐domain engagement scores were identified for each recording site (Table 3). Key moderately associated findings were at F7 (serotonin) and Fz (endorphin), suggesting sensitivity of regional QEEG activity to task‐related emotional and executive engagement demands.

**Table 2:** Engagement Intensity Scores by Activity Domain.

Activity Domain	Engagement Intensity Score (%) with BMP	Engagement Intensity Score (%) with No BMP
Reward‐related	75.61±8.57	79.95±9.86∗
Emotion‐regulation	56.34±15.31	57.36±13.88∗
Social‐affiliation	55.13±13.54	58.09±13.59∗
Pleasure‐oriented	58.56±13.85	55.74±14.27∗

∗p <0.05 refers to paired comparison between BMP and No BMP conditions.

**Table 3:** QEEG Site‐Specific Correlations with Activity‐Domain Engagement Scores.

QEEG Feature	Functional Role	Key Correlation
Fp1 Theta/beta	Anterior prefrontal – calm attentional control & self‐monitoring	Emotion‐regulation (r = 0.419)
Fp1 Beta	Anterior prefrontal – task engagement & dopaminergic alertness	Reward‐related (r = 0.440)
F7 Theta/beta	Left dorsolateral prefrontal – emotional inhibition & top‐down regulation	Emotion‐regulation(r = 0.511)
F7 Beta	Left frontal – affective effort, inhibition, and mood regulation	Pleasure‐oriented(r = 0.419)
F4 Beta	Right dorsolateral prefrontal – working memory, executive planning	Reward‐related(r = 0.418)
Fz Beta	Midline frontal – cognitive control, conflict monitoring, set shifting	Reward‐related(r = 0.454)Pleasure‐oriented(r = 0.500)
Cz Beta	Sensorimotor cortex – motor planning, procedural learning, habit formation	Pleasure‐oriented(r = 0.450)
T3 Beta	Left temporal – auditory‐linguistic integration, procedural memory, social‐affective processing	Emotion‐regulation(r = 0.434)Social‐affiliation(r = 0.432)

As shown in Table 2, engagement intensity scores were consistently higher in the unmonitored (No BMP) condition compared with the BMP‐monitored condition across all activity domains. The relatively large standard deviations indicate substantial inter‐individual variability in task performance and activity prioritization among participants.

Additional correlations revealed distinct roles of each frontal QEEG site in B2B performance. Fp1 theta/beta ratios showed positive associations with serotonin‐linked calmness, while F7 and F4 frontal beta power positively correlated with three neurotransmitter‐linked phases (serotonin, endorphin, and dopamine), suggesting emotional and executive inhibition. Fz and Cz also demonstrated midline involvement in dopaminergic reward and endorphin processing. Oxytocin and serotonin showed low correlations with beta activity at T3.

## 4. Discussion

The B2B protocol, when implemented with BMP monitoring, was associated with engagement of multiple executive function components through digitally mediated, culturally relevant occupational tasks. The longer task durations observed under monitored conditions, particularly during the emotion‐ and memory‐related phases, suggest that real‐time neurofeedback may have increased cognitive effort and self‐regulatory demands during task performance [7, 8]. Consistent with occupational therapy perspectives that emphasize internal cognitive readiness for participation, these findings support the role of BMP as a neurofunctional interface within task‐based rehabilitation rather than as a performance optimization tool [1, 16].

The observation that Engagement Intensity Scores were higher in the unmonitored condition indicates that real‐time QEEG monitoring may impose additional cognitive load during task performance. The presence of monitoring cues and neurofeedback may increase attentional demand, act as a distractor or mild stressor, slow task execution, or heighten self‐monitoring, resulting in lower immediate task efficiency despite potentially deeper cognitive engagement. From an occupational therapy standpoint, such effects are not necessarily unfavorable, as increased cognitive effort and self‐regulation may contribute to longer‐term learning, executive control, and adaptive performance rather than rapid task completion alone.

The relatively large variability observed across QEEG indices and Engagement Intensity Scores likely reflects the clinical heterogeneity characteristic of schizophrenia, including differences in symptom profiles, executive functioning, motivational states, and illness chronicity. High inter‐individual variability is commonly reported in rehabilitation research involving schizophrenia populations and underscores the importance of individualized, functionally informed assessment approaches rather than reliance on uniform performance benchmarks.

Neurophysiologically, frontal theta activity —particularly at Fz and Cz—was consistently elevated during monitored conditions. These regions are associated with working memory, attentional control, and emotional self‐monitoring, and their activation aligns with prior QEEG findings in schizophrenia demonstrating disrupted yet compensatory theta engagement during executive task performance [2, 20]. Analysis of the theta/beta ratio provided additional insight, with increased beta activity during memory‐linked phases suggesting greater executive inhibition and motor planning demands [21].

Interpretation of beta‐band activity warrants a nuanced perspective beyond a single functional role [22]. While increased frontal beta power has traditionally been associated with executive inhibition and cognitive control, emerging evidence suggests that beta oscillations also reflect dynamic modulation of attentional effort, sensorimotor integration, and task‐related readiness. Recent experimental studies have demonstrated that targeted modulation of beta frequency activity—such as through binaural beat stimulation—can enhance task performance, motor coordination, and recovery responses in complex motor and cognitive contexts. These findings indicate that beta activity may index adaptive engagement rather than solely inhibitory processes.

Within the present study, elevated beta activity observed during memory‐ and executive‐demand phases may therefore reflect increased cognitive effort, task monitoring, and preparatory control under conditions requiring heightened self‐regulation. This interpretation aligns with recent evidence showing that externally modulated beta frequencies can facilitate performance and recovery by optimizing neural efficiency and attentional focus [22]. From an occupational therapy perspective, such beta modulation may represent a neurofunctional marker of active task engagement and regulatory effort during complex, goal‐directed occupational performance, rather than a purely inhibitory signal.

The observed delay in performance during monitored tasks further supports the interpretation that BMP feedback modulates cognitive engagement by activating mechanisms related to attentional shifting, inhibition, and emotional control. This pattern is consistent with previous rehabilitation research using routine‐based and remediation‐oriented tasks (e.g., brushing, eating, washing), which emphasize gradual development of attention, flexibility, and sequencing rather than speed or efficiency [10, 12].

Importantly, the integration of B2B and BMP reflects Thai models of rehabilitation [5], such as the Neuro‐Functional Approach (NFA), which conceptualize recovery as a graded process supported by culturally relevant occupation, affective attunement, and structured challenge [15]. By simulating everyday activities in an emotionally resonant and structured digital format, the B2B application aligns with occupational therapy principles of motivational readiness and ecological validity, which are central to effective occupational engagement [13, 17].

From a clinical perspective, the combined use of B2B and BMP offers dual informational value [5]: it captures observable performance‐based indicators (e.g., task duration and success) while also providing neurophysiological indices related to executive strain and affective readiness. In psychiatric occupational therapy, where conventional assessments may inadequately reflect real‐world functioning, such an integrated approach may support more nuanced, individualized clinical reasoning [2, 3]. However, the observed associations between task phase and QEEG activation should be interpreted as exploratory neurofunctional indicators rather than validated digital biomarkers.

Further research is warranted to examine the predictive and longitudinal utility of this approach, including its potential role in therapy monitoring, motivation enhancement, and executive rehabilitation over time. By combining culturally embedded occupational tasks with neuroscience‐informed assessment, the integration of B2B and BMP represents a promising, yet exploratory, direction for occupational therapy interventions in schizophrenia [5].

Several methodological considerations warrant discussion. The complexity and non‐linearity of electroencephalographic signals suggest that advanced analytical approaches, including ML/DL, may uncover latent neurofunctional patterns not readily detectable using traditional inferential statistics. In the present study, the use of theory‐driven, site‐specific metrics facilitated clinically meaningful interpretation aligned with occupational therapy practice; however, this approach may have limited sensitivity to more complex multivariate interactions. As highlighted in recent methodological reviews, ML/DL frameworks are particularly suited for large‐scale, predictive, and longitudinal analyses. Accordingly, future investigations building on this work should integrate ML/DL approaches to enhance robustness, scalability, and predictive insight in digital neurofunctional assessment [9, 10].

Individual alpha peak frequency (IAF) was not explicitly controlled for in the present analysis. Given that IAF can vary in individuals with schizophrenia and may influence the spectral boundaries of theta and beta bands, this represents a methodological limitation. The use of fixed frequency bands may therefore have introduced inter‐individual variability in QEEG measures. Future studies should consider incorporating individualized frequency band adjustments based on IAF to improve sensitivity and neurophysiological specificity.

## 5. Conclusion

The findings demonstrate that QEEG‐based monitoring during occupational tasks can reveal distinct patterns of executive, motor, and emotional engagement in individuals with schizophrenia. Frontal theta and beta activity patterns were differentially associated with neurotransmitter‐linked behavioral phases, underscoring the potential of brain mapping performance (BMP) as a neurofunctional assessment tool. This supports the integration of real‐time neurophysiological feedback into occupational therapy to tailor interventions that enhance emotional regulation, cognitive flexibility, and motivational readiness. These findings should be interpreted as exploratory neurofunctional indicators rather than validated digital biomarkers, pending further longitudinal and predictive validation.

## Author Contributions

Supalak KhemthongK. developed the B2B app and conducted the study. Maliwan RueankamR. supported data analysis and interpretation. Winai ChatthongC. provided project supervision and manuscript oversight as corresponding author.

## Funding

Electricity Generating Authority of Thailand (EGAT).

## Conflicts of Interest

The authors declare no competing interests.

## Data Availability

The data are not publicly available due to privacy and ethical restrictions related to participant confidentiality.
